# Role of Co-creation for Large-Scale Sustainable Adoption of Digitally Supported Integrated Care: Prehabilitation as Use Case

**DOI:** 10.5334/ijic.6503

**Published:** 2022-10-07

**Authors:** Erik Baltaxe, Isaac Cano, Raquel Risco, Raquel Sebio, Fernando Dana, Sara Laxe, Ramon Martínez, Fernando Ozores, Josep Roca, Graciela Martínez-Pallí

**Affiliations:** 1 Institute of Pulmonology, Physiology and Exercise. Sheba Medical Center, Tel-Hashomer, Ramat-Gan, Israel; 2 Institut d’Investigacions Biomèdiques August Pi i Sunyer (IDIBAPS), University of Barcelona (UB), Villarroel 170, 08036, Barcelona, Catalonia, Spain; 3 CIBER of Respiratory Diseases (CIBERES), Madrid, Spain; 4 Anesthesiology Department. Hospital Clínic de Barcelona Villarroel 170, 08036, Barcelona, Spain; 5 Physical Medicine and Rehabilitation Department. Hospital Clínic de Barcelona, Villarroel 170, 08036, Barcelona, Spain; 6 Stimulo Design SL, Plaça Damià Mateu 1, 08450, Llinars del Vallés, Catalonia, Spain; 7 Buenaidea Creatividad & Innovación SL, Aragó 184, 08011, Barcelona, Catalonia, Spain; 8 Institut Clinic Respiratori. Hospital Clínic de Barcelona, Villarroel 170, 08036, Barcelona, Catalonia, Spain

**Keywords:** co-creation, prehabilitation, physical activity, nutritional optimization, mindfulness, mHealth

## Abstract

**Introduction:**

The efficacy-effectiveness gap constitutes a well-known limitation for adoption of digitally enabled integrated care services. The current report describes the co-creation process undertaken (2016–2021) to deploy a prehabilitation service at Hospital Clínic de Barcelona with the final aim of achieving sustainable adoption and facilitate site transferability.

**Methods:**

An implementation research approach with a population-based orientation, combining experience-based co-design and quality improvement methodologies, was applied. We undertook several design-thinking sessions (Oct-Nov 2017, June 2021 and December 2021) to generate and follow-up a work plan fostering service scalability. The implementation process was assessed using the Comprehensive Framework for Implementation Research, leading to the identification of key performance indicators.

**Discussion:**

Personalization and modularity of the intervention according to patients’ surgical risk were identified as core traits to enhance patients’ adherence and value generation. A digitally enabled service workflow, with an adaptive and collaborative case management approach, should combine face-to-face and remotely supervised sessions with intelligent systems for patients’ and professionals’ decision support. The business model envisages operational costs financed by savings generated by the service.

**Conclusions:**

Evidence-based co-creation, combining appropriate methodologies and a structured evaluation framework, was key to address challenges associated with sustainable prehabilitation service adoption, scalability and transferability.

## Abstract (ca)

**Introducció:**

La bretxa eficàcia-efectivitat limita l’adopció de serveis d’atenció integrada amb suport digital. L’estudi descriu el procés de co-creació efectuat (2016–2021) per desplegar, a l’Hospital Clínic de Barcelona, un servei de prehabilitació de pacients de risc per a procediments quirúrgics, amb l’objectiu d’aconseguir una adopció sostenible del servei i facilitar-ne la transferibilitat.

**Mètodes:**

Es van aplicar eines de recerca d’implementació amb una orientació poblacional, combinant metodologies de codisseny basades en l’experiència i de millora de la qualitat. Es van realitzar diverses sessions de design-thinking (Octubre-Novembre de 2017, Juny de 2021 i Desembre de 2021) per generar, i fer el seguiment, d’un pla de treball concebut per assolir escalabilitat del servei. El procés d’implementació es va avaluar utilitzant el Consolidated Framework for Implementation Research (CFIR), que va conduir a la identificació d’indicadors clau de rendiment.

**Discussió:**

La personalització i la modularitat de la intervenció segons el risc quirúrgic dels pacients es van identificar com a trets bàsics per millorar l’adherència i la generació de valor. La organització de la prehabilitació, amb un enfocament adaptatiu i col·laboratiu de gestió de casos, hauria de combinar sessions presencials i supervisades remotament amb sistemes intel·ligents de suport a la decisió per a pacients i professionals. El model de negoci preveu que els costos operatius de la prehabilitació siguin finançats per l’estalvi generat.

**Conclusions:**

El procés de co-creació, combinant metodologies adequades i un marc d’avaluació estructurat, va esser clau per abordar els reptes associats a l’adopció sostenible del servei, així com la seva escalabilitat i transferibilitat.

## Introduction

Evidence-based benefits of a clinical intervention demonstrated in a highly controlled setting (efficacy) very often cannot be generalized to the real-world scenario (effectiveness) within the same site. The phenomenon, known as efficacy-effectiveness gap (EEG) [1,2], is one of the major obstacles to demonstrate health value generation, and to achieve sustainable adoption, of integrated care services [3,4,5]. Likewise, overcoming EEG challenges is crucial for successful transferability of the results across heterogeneous sites. One of the proposed implementation mechanisms to optimize large-scale deployment and adoption of integrated care is to undertake an early process of co-creation with input of key stakeholders [4]. Expected outcomes of such process are service workflow co-design leading to healthcare value generation.

The current report summarizes the process of co-creation and adoption of prehabilitation [6,7] as a mainstream integrated care service at Hospital Clinic de Barcelona (HCB) during the last five-year period, from its initial piloting in mid-2016 [8,9] throughout its mature implementation until its readiness for transferability in 2021 [10].

Prehabilitation is defined as a patient-tailored preoperative short-term intervention, four weeks on average, encompassing, but not limited to: exercise training, promotion of physical activity, nutritional optimization and psychological support. Enhanced management of multimorbidity and prevention of unhealthy habits are also tackled. The final aim of prehabilitation is to improve functional capacity of patients undergoing elective major surgery as an attempt to minimize postoperative morbidity and accelerate recovery [6]. It is envisaged as a preventive standard clinical practice to be included into Enhanced Recovery After Surgery (ERAS) programs [11,12,13].

The primary aim of the Prehabilitation Unit at HCB Unit is to cover the needs generated by high-risk candidates to several major surgical procedures. However, the combination of progressive improvements in longevity, coupled with the increasing prevalence of multimorbidity with age, has resulted in a growing number of surgical procedures taking place in elderly patients with co-existing medical conditions. Since postoperative complications, particularly in this population, constitute a major burden on health systems, there is a need for a population-based approach of perioperative care [7]. Accordingly, an additional aim of the Unit is to foster a population-based approach to personalized prehabilitation covering all surgical risk strata in the HCB reference area.

Whereas prehabilitation for high surgical risk patients can benefit from ad-hoc digital support to enhance interdisciplinary coordination among different in-hospital services implicated the intervention (anesthesia, surgery, rehabilitation, nutrition, psychology); a population-health approach requires prehabilitation to be a digitally-enabled integrated care service by-design, with participation of different community-based stakeholders (i.e., primary care professionals and health coachers based in sports clubs). Consequently, there was a clear need for a co-creation process toward refinement of the standard prehabilitation intervention to build capacity, increase healthcare efficiencies and foster transferability to other sites within the frame of the EIT Health innovation action PAPRIKA [10,14].

The objective of the current manuscript is to describe the co-creation process undertaken during 2017–2021 to pave the way for large-scale adoption of prehabilitation with a population-based approach.

## Ethical Approval

The Ethics Committee for Clinical Research at HCB approved the study (HCB/2016/0883). The interviews were recorded. Informed consent was understood, accepted and signed by all patients and caregivers. The study was registered at ClinicalTrials.gov [NCT02976064 – Implementation of Collaborative Self-management Services to Promote Physical Activity (NEXTCARE-PA)].

### Description of the Care Practice

Prehabilitation at HCB builds on prior evidence of efficacy and its potential for cost-effectiveness in high-risk patients undergoing major digestive surgery generated through a randomized controlled trial (RCT) during the period 2013–2016 [8,9]. Prehabilitation added costs to the surgical process, but this was offset by reduction of complications, shorter ICU hospital stay and reduced early re-admissions rates after hospital discharge. Following these encouraging results, the prehabilitation service was deemed ready for implementation as mainstream service at HCB, leading to the creation of the Prehabilitation Unit in 2016 and to the initiation of the current implementation research process.

### The entire co-creation period

**Figure 1:**
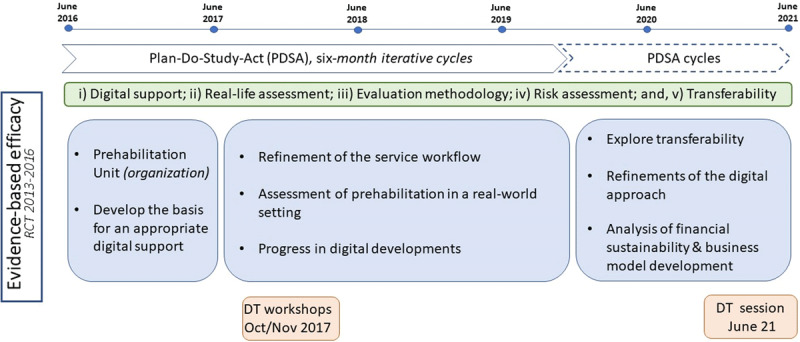
Timeline for co-creation and adoption of prehabilitation at HCB. Distribution of tasks through the experience-based co-design and quality improvement implementation research process; DT: Design-Thinking.

The core objectives of the co-creation process experienced a clear evolution summarized in three consecutive phases depicted in Figure 1. The first year was devoted to the organization of the Prehabilitation Unit and to develop the basis for an appropriate digital support to the service. During the subsequent period, until end-December 2019, main achievements were refinement of the service at HCB, and assessment of the activity of the Prehabilitation Unit following the evaluation framework described in [15]. The activities undertaken during the last eighteen months, starting at January 2020, had a threefold objective: transferability analysis, achievement of digital maturity and to assess financial sustainability.

The co-creation process was initially focused on adoption of the service at the Integrated Health District of Barcelona-Esquerra, 520 k citizens [16], falling within the activities of the Catalan Open Innovation Hub on Digitally-Enabled Integrated Care Services, one of the four original EU Good Practices in [14]. As such, the deployment strategies reported in the current document were fully aligned with the Catalan Health Plans 2011–2015 [17] and 2016–2020 [18], promoting digitally enabled integrated care. It is of note that the tasks reported have been developed under the umbrella of complementary EU projects [10,19,20,21] addressing different facets, all of them converging toward optimization of digitally-enabled integrated care.

During the initial forty two months period, from mid-2016 to end-2019 (Figure 1), a systematic quality improvement approach using iterative 6-month Plan-Do-Study-Act (PDSA) cycles [22,23,24] was implemented with a twofold purpose: i) to generate the service workflow design of the interventions associated to the two case studies addressed in [19,20], one of them being prehabilitation; and, ii) to guide the digital developments supporting the target integrated care services with an adaptive and collaborative case management approach [25,26]. This period was followed by a second co-creation phase, with a more informal PDSA approach, focused on refinement and fine-tuning of the digital tools (end-2019 to mid-2021). It should be highlighted that in the analysis of the prehabilitation service, five dimensions were taken into consideration, as reported in [19,20]: 1) Analysis of deployment in real-life scenarios; 2) Digital support; 3) Health risk assessment and service selection; 4) Evaluation Methodology; and, 5) Transferability and site adoption.

The co-creation process (Figure 1) contributed to consolidate prehabilitation at HCB as a standard service for approximately 150 candidates per year undergoing major surgeries in different specialties, namely: digestive, cardiac, thoracic, urologic and gynaecologic. It is of note that the capacity of the prehabilitation unit covered less than 20% of the estimated demand, mainly due to the limited capacity of the exercise training facilities at HCB. This aspect, together with patient’s logistic/accessibility limitations, prompted two types of multimodal prehabilitation programs: i) a physical activity (PA)-based program; and ii) a face-to-face supervised exercise training (ET)-based program, with low and high requirements of human/logistic resources, respectively.

Apart from enhanced management of multimorbidity and prevention of unhealthy habits, the PA-based program included: i) motivational interviewing; ii) a physical activity promotion plan; iii) nutritional optimization; and iv) psychological support.

On the other hand, the ET-based program included all the elements of the PA-based program and, additionally, hospital-based face-to-face supervised exercise training sessions two-three times per week. The ET-based program was prioritized for patients with significant multimorbidity and patients with physical deconditioning undergoing highly aggressive surgeries.

As indicated above, the co-creation process covered five dimensions (i-v) depicted in Figure 1. The analysis of the prehabilitation results in a real-life scenario at HCB was undertaken for a thirty-month period, from mid-2017 to end-2019, as part of the evaluation framework described in [15]. It is of note that PDSA cycles played a major role in the entire quality improvement approach also contributing to feed the Design Thinking sessions. The debates generated during the two initial PDSA cycles consolidated the need for development, adoption/adaptation, of interoperable digital tools providing functional and technological integration with different healthcare providers. The team conceptualized the need for covering three differentiated, though intertwined areas, with specific technological requirements: i) patients’ accessibility and empowerment; ii) enhanced management of care paths; and iii) collaborative work between two or more stakeholders (patient/carers and professionals), eventually from different healthcare tiers/providers. The specificities of the technological requirements to be operational on top of existing health information systems were explored, and developed, during the study period. Achievements in the other three dimensions considered in the co-creation process (Figure 1): Health risk assessment and service selection; Practicalities of the implementation of the evaluation framework [15]; and, Analysis of transferability and site adoption are summarized below, as part of the description of the Design Thinking sessions, as well as under the subheading on large scale sustainable adoption.

### PDSA cycles

Periodical meetings in a monthly basis were held throughout the PDSA cycles. Technologically oriented meetings (the last Thursday of the month) included three professionals with technological profile and seven persons with clinical background. All of them pertaining to the research team. Controversial and strategic aspects were further discussed and decided in the scientific meetings (the last Friday of the month) carried out by a core subset of six professionals with technological and clinical backgrounds. It is of note that patients’ inputs were captured with regular interviews and surveys on specific aspects of the service workflow and technologies used. However, informal patients’ feedback to health professionals was feeding the co-creation process throughout the entire study period. Moreover, we stimulated synergies between the clinical teams delivering prehabilitation and the technological partners developing the digital tools.

The approach aimed to provide overview, ownership, and involvement of stakeholders on the intervention processes, while encouraging management responsibilities to ensure focus, pace, and self-discipline in the process. Moreover, the pragmatic nature of the adopted PDSA methodology provided flexibility to develop interventions according to stakeholder’s feedback ensuring fit-for-purpose solutions, while providing the opportunity to build evidence for change and engage stakeholders as confidence in the intervention increased. The multidisciplinary composition of the co-creation teams at site level aimed to facilitate a good understanding of the complex interactions among multiple non-technological factors, internal and external, that modulate adoption of digitally enabled integrated care services in real life settings.

### Design-Thinking sessions

The co-creation process included experience-based co-design and quality improvement process in the form of several Design Thinking (DT) sessions [27,28,29,30,31] which were carried out during October-November 2017; on 22^th^ June 2021; and, on 13^th^ December 2021. While 2017 encompassed three sessions assessing the service in a comprehensive manner [32], 2021 encompassed two sessions focused on the specificities of the interplay between the hospital-based prehabilitation team and professionals from different collaborating sports centres in the city of Barcelona, highly encouraged in the conclusions of the 2017 DT sessions. Main traits were as follows:

***2017 Design-Thinking (DT) sessions*** – Were preceded by a **Preliminary fieldwork** analysis with the surveys done to professionals and patients. It contributed to define the characteristics of the three DT sessions, as displayed in Table 1 wherein objectives, tools and results of each session are summarized. A detailed description of the design-thinking sessions can be found in **Section 1** of the on-line supplementary material. Three DT sessions, each of a four-hour duration, aiming to address the core aims of the study, were carried out. Core objectives of the workshops were: i) to identify actionable factors modulating regional scalability of prehabilitation; ii) to enhance efficiencies of the service with the use of digital tools, and, iii) to design a business model contributing to sustainable adoption of the service. The final goal was to generate a roadmap to foster regional scalability of prehabilitation in Catalonia (ES) (7.7 m citizens).

**Table 1:** Objectives, tools and main results of the three 2017 design thinking sessions.


	AIMS	TOOLS	RESULTS

**Preliminary fieldwork**	To capture the patient experienceperspective of the service. To identify factors of the prehabilitationservice at HCB that may limit scalability.	In-depth interviews to patients and caregivers. Surveys to professionals involved in the prehabilitation unit.	Identification of actionable areas to be addressedin Session I – Immersion (**see text**).

**IMMERSION** *(Session I)*	To gain further insight on organizational and actionable factors of to enhance scalability of the existing prehabilitation to: Optimize service workflow. Identify ICT-support to scalability. Explore financial needs for adoption.	Elaboration of the following material contributing to refinementof the PreHab service (*): Experience map Empathy map Context map Priority map	Agreement on the main challenges to face and solve in Sessions II and III. Main outcome of the Immersion was **“to *provide an accessible, round-the-clock personalized and modular service that the patients should be ableto use autonomously during the PreHab period. The service should combine remotely controlled actions and face to face interactions with health professionals”.***

**IDEATION** *(Session II)*	To generate, develop and assess ideas and plans to solve the challenges identified in Session I.	Two inspirational presentations (**see text**). Small groupcreative sessions. Positioning map (*)	Generation of a customer journey thatshould contribute to define a viable strategy for regional deploymentof prehabilitation. To this end, an overview of the prehabilitationservice workflow was produced, as a visual map depicting theend users touch points and needs for both ICT-supportand business model.

**VALIDATION** *(Session III)*	To consolidate theproposals and refine the actions resulting from Session II aimingto define a viable strategy for regional deployment of arefined service workflow.	Three working groups to separately tackle specific areas and final overall group meeting to generate consensus on specific proposals for each area: ✔ Implementation strategies. ✔ Technology-related aspects. ✔ Business model & reimbursement incentives.	Fulfil end-user touch points (**see text for more details**) Creation of a capillary network of healthcare/wellness centres to enhance accessibility. Mobile app fostering tailored patient empowerment for self-management and remote monitoring. Interoperability of ICT-enabling tools with existing HIS. ACM system to support prehabilitation knowledge intensive processes for enhanced service management. To drive patient interactions and data collection through an AI assisted chat *(i.e. Chatbot)*. Cost-savings generated by prehabilitaton should cover the operational costs of the service. Investments needed to launch the service, as well as reimbursement incentives, could be covered by innovative PPP models.


HCB: Hospital Clínic de Barcelona; ICT: Information and communication technologies; HIS: Hospital Information Systems; ACM: Adaptive case management; AI: Artificial intelligence; Chatbot: A computer program designed to simulate conversation with human users, especially over the Internet; PPP: public-private procurement; (*) See description in on-line supplementary material.

The content of the three DT sessions covering: Immersion, Ideation and Validation (Sessions I-III, respectively), was based on preliminary work consisting of two actions. Firstly, we performed a survey aiming at gaining insight into the organizational aspects of the prehabilitation structure (Prehabilitation Unit) and service workflow at HCB. The survey was carried out with professionals involved in the design and management of the service. It also included other healthcare professionals having direct contact with the patients enrolled in the service, namely: anaesthesiologists (n = 5), physiotherapists (n = 3), nurses (n = 10), nutritionists (n = 2), psychiatrists (n = 2) and psychologists (n = 2). Secondly, we carried out in-depth face-to-face interviews with five patients and their respective caregivers who had participated in prehabilitation, aiming at capturing the patient experience perspective of the service. Patients surveyed in this phase had been candidates for cardiac transplantation, resection of lung parenchyma or major abdominal oncological surgery. It is of note that the additional collaborative methodology applied in [19] including patients’, professionals’ and managers’ surveys, generated input material for the DT sessions.

The three DT sessions included all the stakeholders’ profiles, namely: healthcare professionals (n = 13), managers (n = 3), designers (n = 6), health-technology agents (n = 3), business school representatives (n = 2), innovation agents (n = 10) and policy makers (n = 2) (sessions’ details are reported in Table 1S).

The first session, Immersion, contributed to identify several different factors with potential impact on the service scalability. The most relevant ideas were clustered into the three dimensions: i) Users’ satisfaction; ii) Technological viability; iii) Economic viability that were identified as key areas of action to foster prehabilitation scalability and adoption. It was agreed that actions should converge toward the service definition depicted in Table 1 (second row, third column). Overall, five areas for action were formulated: i) Personalization of interventions based on surgical risk assessment among other factors; ii) Stimulation of a pro-active role of patients, aiming at empowerment for self-management and promotion of physical activity; iii) Enhanced flexibility of interventions through a highly modular service design, facilitating service personalization; iv) Improved accessibility and logistics; and, v) Achievement of financial sustainability of the services to ensure long-term adoption of cost-effective healthy lifestyles interventions.

The second session, Ideation, was initiated with a short inspirational presentation, 10 min, to update the audience on the status of the prehabilitation service. A second talk, 15 min, was geared towards exploring previous experiences in other fields that have solved similar challenges. It was followed by ten simultaneous small group creative sessions, 4–5 persons each, that approached the main previously identified challenges under the following success criteria: i) Allow scalability while preserving the quality of the service; ii) Allow reproducibility of the service outcomes in different sites, that is, service transferability; iii) Enhance the adherence of patients to the work plan; iv) Provide key performance indicators to track service effectiveness; v) Foster accessibility to the program; vi) Ensure economic viability for sustainability; and, vii) Conceive the service within a LEAN approach [33,34] to allow agile implementation and management using minimal resources. The ideas resulting from the creative sessions were debated by the whole group and then prioritized and pooled into a positioning map. Finally, the ideas incorporated in the positioning map were used to generate a general overview for the refined prehabilitation service workflow to be assessed during the third session, Validation. The categories displayed in the priority map were further debated and elaborated in three subgroups of attendees: i) group A: End-user touch points; ii) Group B: Digital tools; and, iii) Group C: Business, to achieve a well-defined action plan for scalability of the service, as summarized in Table 1 (fourth row, third column).

***2021 Design-Thinking sessions*** – Two three-hour sessions carried out on 22^nd^ June and 13^th^ December 2021 involved core members (on average 18–20 persons in each session) of the clinical prehabilitation team, representatives of three different sports centers and technological experts of three digital small and medium enterprises (SME) and one technological institute. The focus of the June session was on the design of operational aspects of the interplay among the hospital-based team, collaborating sports centers and primary care health professionals.

The DT session held on 22^nd^ June 2021 was focused on the design of pilot study to explore patient acceptability and practicalities of the interplay between the hospital-based team and different sports centres willing to collaborate to increase the weight of community-based execution of the program, as well as to generate a population-based approach to prehabilitation.

The final DT session on 13th December evaluated preliminary data of a two-month pilot experience partly transferring the intervention to sports centres. Two main outcomes were confirmation of feasibility and proposal of a three-layer service design covering the entire spectrum of patient’s risk. Accordingly, the service is being organized as follows. i) low risk patients are candidates for an educational intervention and remotely supported behavioural change; ii) patients situated at the medium risk layer are also candidates for promotion of daily-life physical activity and community-based, partly remotely supported, physical training; and iii) high risk patients add to the previous two levels of intervention an initial period with hospital-based face-to-face supervised high-intensity exercise training followed by community based physical training. The December DT session confirmed the potential for transferability aiming at launching the community-based prehabilitation service during the first quarter of 2022.

### Large-scale sustainable adoption

The process of implementation of prehabilitation during the study period was assessed using the Consolidated Framework for Implementation Research (CFIR) [35]. Moreover, in the initial phase, we evaluated the ecosystem maturity for digital transformation and deployment of integrated care services using the Scirocco Maturity Model for Integrated Care [36].

The CFIR information was grouped in five different areas, namely: i) Intervention characteristics; ii) Outer setting; iii) Inner setting; iv) Characteristics of the individuals; and, v) Characteristics of the process. It is of note that lessons learnt from CIFR, as well as knowledge from existing literature [8,9,37,38,39], were useful to identify key performance indicators (KPI) for the program long-term follow-up after adoption.

The implementation process following the five items of the CFIR approach [35] is summarized below (Table 2) and in Figure 1 (co-creation process). Briefly:

**Table 2:** Implementation of prehabilitation at HCB, KPI and recommendations for scaling-up.


CFIR CONSTRUCTS	CFIR MAIN POINTS	KEY PERFORMANCE INDICATORS	CHALLENGES & RECOMMENDATIONS

**Intervention** **Characteristics**	– Prehabilitation as an integrated care component of ERAS pathways (enhanced recovery after surgery) – Core components: Management multimorbidity Trimodal intervention Service workflow defined Define target patients’ profiles Personalize the service – Adaptability of non-core components is required – Continuous quantitative & qualitative build-in evaluation is needed	**STRUCTURE** **Coverage** **PROCESS** **Rate of dropouts** **Rate of adherence** **Quality assurance scoring** **POST-OPERATIVE OUTCOMES** **Comprehensive Complications Index** **Hospital length of stay** **Use of healthcare resources at 30 days**	Increase service efficiency & value Building capacity & Refinementof service delivery Enhanced risk assessment & program prescription Improving digitalsupport Transfer to the community

**Outer Setting**	– Patient-centred orientation, a core trait – Networking across experiences needed – Site customization is required to minimize potential negative impacts of external factors		

**Inner Setting**	– Bottom-up/Top-down interactions are needed for success. Champion driven programs show high success rates – Key resources togenerate/reinforce a positive climate change are needed		

**Characteristics of Individuals**	– Continuous monitoring of satisfaction levels and consideration of feedbackfrom patients and professionals is highly recommended		

**Process**	– A building-blocks implementation strategy, with appropriate site customization prioritizing engagement, is required – Continuous evaluation of results		


*Intervention characteristics*: We identified modularity and personalization of the prehabilitation program as key attributes of the service which will influence the success of implementation. However, the following core components of the program must be acknowledged: (i) High-intensity exercise training; (ii) Promotion of physical activity; (iii) Nutritional support; (iv) Behavioural intervention, as reported in [8,9]. Besides that, the program will also require the adaptability of non-core components such as psychological support, smoking cessation programs and haemoglobin optimization, among others.

Another key aspect for a successful implementation of prehabilitation programs is an enhanced logistics and better health risk assessment. These components will not only lead to early identification of candidates for prehabilitation but also it will enhance the personalization of the interventions included in each patient work plan.

The evolution toward a community-based service to overcome the current constraints of prehabilitation (i.e., limited capacity of hospital facilities, convenience of facilities closer to patients’ residency, efficiencies of care continuum) is cornerstone to achieve service scalability and transferability. However, quality standards of the intervention should be maintained. Finally, the importance of a continuous quantitative & qualitative build-in evaluation of the prehabilitation service, using well-identified KPI, must be highlighted. Transition from a hospital-based intervention to a community-based delivery of prehabilitation was planned during the 2021 DT sessions and currently assessed through a pilot program.

*Outer setting* – We understand that a patient-centred orientation considering patients’ preferences, facilitators and barriers, should be a core trait of the prehabilitation program. Moreover, although clinical site customization is required, networking across different prehabilitation experiences enriches the programs.

*Inner setting* – Bottom-up & top-down interactions are needed for a successful implementation of the service. Moreover, key resources to generate and reinforce a positive climate change within the Institution are needed.

*Characteristics of the individuals* – There is a need to stress continuous monitoring of satisfaction levels. Consideration of feedback from patients and professionals is highly recommended. In that sense, PDSA cycles, DT sessions and focus groups are interesting tools to introduce for the guiding of the implementation process.

*Characteristics of the process –* We recommend facing the implementation process of a modular prehabilitation programs within a building-blocks strategy. This implementation approach will facilitate site customization and will also help to prioritize the engagement. Moreover, we also recommend the continuous evaluation of results during this process. As mentioned, elaboration and follow-up of an appropriate Quality Assurance program is a must.

It is of note that the Scirocco assessment indicated a high level of maturity of the Health District for adoption and further evolution of the prehabilitation service [40].

### Quality assurance in a real-world scenario

The evaluation of the prehabilitation service in a real-life setting at HCB during a thirty-month period, from mid-2017 to December 2019, as well as existing literature [6,7,8,9], provided the basis for proposing KPI structured using the Avedis Donabedian’s model [41], as indicated in Table 2, second column.

Future validations of the proposed KPI in real-life settings should facilitate continuous quality assessment of the service using user-profiled dashboards, useful for clinical and administrative management of the service, aiming at optimization clinical outcomes and/or value generation of the prehabilitation. Cost-consequence analyses done using data from the reported RCT [8,9] and from assessment of the service in a real-life setting [42] strongly indicate financial sustainability of prehabilitation in high-risk patients paid by healthcare providers. However, delivery of the service in low and medium risk candidates deserves further studies.

## Discussion

The current study addressed major prehabilitation service challenges for large-scale sustainable adoption of the intervention, through a co-creation process that used experience-based co-design tools to identify key elements to be considered for regional scalability and site transferability. Other priority areas also being addressed, but not described in the current report, were: i) Continuous quality improvement of the service in real world settings, aiming at ensuring long-term reproducibility of the initial study results; ii) Enhanced risk assessment for personalization of the service; and, iii) Evolution of prehabilitation toward a population-based approach, which implies tailoring the intervention according to a subject-specific health risk assessment, as well as extending the scope of the intervention to also enhance post-surgical care recovery. It is of note that, during the entire study period, we explored the potential for generalization of the approach to other use cases, namely: rehabilitation of chronic patients, including support to oncologic patients, and early prevention of multimorbidity in high-risk citizens.

We believe that service co-creation and adoption based on the combination of experience-based co-design and a quality improvement process facilitated a stepwise progress towards identifying the three pivotal dimensions requiring intervention: i) Enhanced service design; ii) Digital support; and, iii) Financial sustainability. It is acknowledged that site customization of the service will be required for large scale implementation at regional or international levels. Personalization and modularity of the prehabilitation service have been stressed as two core traits needed for successful site implementation. Likewise, empowerment of patients for self-management of their condition constitutes an essential goal of the service. The requirements for digital support in the scalability of prehabilitation have been formulated in detail in [43] and commercial promotion will be initiated within 2021 through the spin-off company Health Circuit [44]. It is of note that the technological support facilitating service modularity and personalization as well as interoperability between community-based facilities, including patient’s home, and hospital-based information systems has been achieved in the health district of Barcelona-Esquerra (520 k inhabitants).

Beyond prehabilitation, we believe that the current study indicates a high potential of co-creation, and DT methodologies, for contributing to the refinement and site adaptation of integrated care service workflows in a broad spectrum of complex interventions as often encountered in the integrated care scenario [40].

## Lessons Learnt

The co-creation process described in the current report allowed to identify the following areas for action aiming at optimizing value generation and large-scale adoption of prehabilitation:

- *Capacity building and refinement of service delivery –* It involves actions on service re-design using a LEAN approach aiming at enhancing patients’ accessibility and adherence, as well as broadening the scope of service delivery to different settings (i.e. health clubs and sport centers), beyond a hospital-centered approach described in the current report.
- *Enhanced risk assessment for personalization of interventions* is needed to facilitate fine-tuning of the three-layer service design described above.
- *Maturity of digital support constitutes a high priority to optimize prehabilitation outcomes* [26,43].
- *Future co-creation initiatives aiming at service refinement should address specific, and narrower, targets to ensure short-term achievements*.

## Conclusions

The current report provides three well-defined outcomes. Firstly, it illustrates the potential of evidence-based co-creation, specifically using DT methods, and quality improvement methodologies with iterative PDSA cycles to achieve large-scale implementation of integrated care services for chronic patients, taking as a use case prehabilitation. As a second outcome, it identified factors influencing prehabilitation results and the determinants of adoption of the service, using the CFIR framework. Finally, from the lessons learnt, we propose a list of Key Performance Indicators for long-term quality assurance of the intervention after adoption. Overall, the co-creation approach shows high potential for service refinement in other complex healthcare interventions.

## Additional File

The additional file for this article can be found as follows:

10.5334/ijic.6503.s1 Supplementary File 1. Design Thinking Sessions and CFIR Description.
