# Supplementary material for: Role of Co-creation for Large-Scale Sustainable Adoption of Digitally Supported Integrated Care: Prehabilitation as Use Case
Source: Int J Integr Care. 2022 Oct 7;22(4):1. doi: 10.5334/ijic.6503 (PMC9541166; doi:10.5334/ijic.6503)
Supplement: Supplementary File 1. — Design Thinking Sessions and CFIR Description. [file ijic-22-4-6503-s1.pdf]

Role of co-creation for large-scale sustainable adoption of digitally supported integrated care: prehabilitation as a use case

(on-line supplementary material)

The current document has three different sections providing detailed information on: i) 2017 Design Thinking (DT) sessions (Section 1A); ii) 2021 DT sessions (Section 1B); iii) Consolidated Framework for Implementation Research (Section 2); and, iv) Key Performance Indicators (Section 3), that complements the main manuscript.

Section 1 - 2017 Design-Thinking (DT) sessions

The content of the three DT sessions was based on preliminary work consisting of two actions. Firstly, we performed a survey aiming at gaining insight into the organizational aspects of the prehabilitation structure (Prehabilitation Unit) and service workflow at Hospital Clinic de Barcelona (HCB). The survey was carried out with professionals involved in the design and management of the service. It also included other healthcare professionals having direct contact with the patients enrolled in the service, including anaesthesiologists, physiotherapists, nurses, nutritionists, psychiatrists and psychologists. Secondly, we carried out in-depth face-to-face interviews with five patients and their respective caregivers who had participated in prehabilitation, aiming at capturing the patient experience perspective of the service. Patients surveyed in this phase had been candidates for cardiac transplantation, resection of lung parenchyma or major abdominal surgery due to cancer. It is of note that the additional collaborative methodology applied in ACT@Scale project [1] including patients', professionals' and managers' surveys, generated input material for the DT sessions that significantly enriched the work described below.

The three DT workshops included all the stakeholders' profiles, namely: healthcare professionals and managers, designers, health-technology agents, business school representatives and policy makers, as described in detail below for each session (**Table 1S**).

Preliminary fieldwork - Briefly, in the preliminary fieldwork, the surveyed professionals recognized the active role of the patients and the personalization of the service workflow, as two key factors for successful completion of the work plan. They insisted on the need for strategies fostering patient empowerment for self-management. Healthcare professionals also highlighted the multidisciplinary nature of prehabilitation as the most valued characteristic of the program. They also stressed the need for complementing face to face supervised activities conducted by physiotherapists with additional community-based activities, as part of the service work plan. It is of note that community-based tasks can be remotely controlled or carried out with face-to-face supervision by professionals working in partnering centres (i.e., sport centres, wellness clubs, etc.). Moreover, the professionals identified a clear need for coordinating the tasks scheduled in the patients' action plan. Accordingly, the use of information and communication technologies (ICT) enabling information sharing, remote off-line control and collaborative work among professionals (hospital-based team, wellness centres, primary care team, etc.) across healthcare tiers was identified as a major issue.

Both patients and caregivers valued their experience very positively. In all cases, the most valued intervention was the exercise training program because patients were able to perceive the improvement of their physical performance and health status.

They positively identified group-based mindfulness sessions, as well as personalization of the intervention, as effective ways to encourage both adherence to the work plan and reduce anxiety. However, they identified as a weakness the limitations of the existing infrastructure (i.e., bigger both gym and locker rooms and showers), which would help to improve the experience. Interestingly, patients indicated the relevance of a future extension of the service during the postoperative period. As mentioned, the outcomes of the two preliminary surveys addressed to professionals and to patients/caregivers, respectively, were used to define both the aims and content of the first session, as described in the main manuscript (**Table 1**).

Table 1S. Stakeholder profiles contributing to the three co-design sessions.

	**IMMERSION (Session I)**	**IDEATION *(Session II)***	**VALIDATION *(Session III)***
**Prehabilitation team**			
Anaesthesiologists	2	2	1
Physiotherapists	2	2	2
Nutritionists	1	1	
Nurses	1		1
Psychiatry			1
Psychologist		1	1
**Healthcare professionals**			
Physicians	2	4	4
General practitioners	1	2	2
Physiotherapists	3	3	2
Nurses	1	1	2
**Designers**	6	6	6
**Innovation agents**	9	10	12
**Health-technology agents**	3	5	3
**Business school representatives**		2	1
**Policy makers**	2	2	2
**Healthcare companies representatives**	3	3	3
**TOTAL ATTENDEES**	**36**	**44**	**43**

Session I: Immersion - A total of 36 attendees, representatives of the different stakeholder profiles, contributed to the first session (**Table 1S**). The experience map

(**Figure 1S**) shows high levels of satisfaction and acceptability of the patients through the overall service workflow. We can observe, however, a high diagnosis-related level of anxiety at service inclusion, progressively decreasing throughout the prehabilitation period. Three main ideas were extracted from the analysis of the experience map of the current service: i) Limitation of resources in terms of space and facilities available, as well as regarding the number of health professionals involved in the service delivery; ii) Effectiveness of the current service provision relies upon a close and personalized face-to-face follow-up of patients which may be detrimental for its future scalability; and last but not least, iii) Additional ICT-support may contribute to generate efficiencies and to

extract metrics for service monitoring.

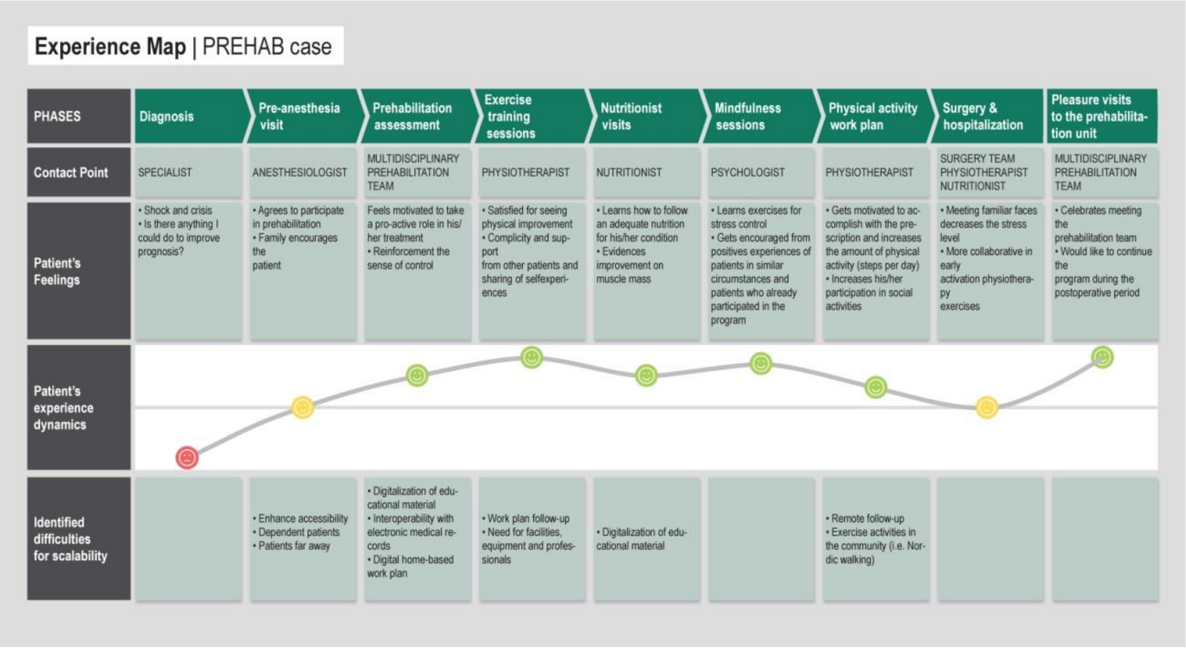

***Figure 1S Patient experience map** - The prehabilitation experience map represents the complexity of the prehabilitation experience while capturing the common points of change and transition throughout the different stages of the perioperative process (columns), namely: i) Diagnosis; ii) Visit to the pre-operative clinic; iii) First visit to the prehabilitation unit and baseline multidisciplinary assessment; iv) Exercise training sessions supervised by a specialized physiotherapist; v) Nutritional follow-up visits by a registered nutritionist; vi) Group-based mindfulness sessions driven by a specialized psychologist; vi) Community-based physical activity*

plan based on increasing the number of steps per day (pedometer); vii) Surgery and hospitalization period; viii) Pleasure visits to the prehabilitation unit.
On the rows we can observe four main domains: i) The contact points of each stage of the prehabilitation process and the representative professional for each contact point; ii) Patient's feelings, thoughts, and actions relative to each stage of the prehabilitation period; iii) A graphical representation of the patient's experience dynamics through the prehabilitation process; and, iv) The difficulties identified in each stage which may be limiting service scalability.

A subsequent brainstorming session, including analysis of the empathy map (**Figure 2S)**, contributed to identify several different factors with potential impact on the service scalability. All the ideas raised in the brainstorming session were pooled in a context map (**Figure 3S**) under the following labels: i) Social and healthcare sector trends; ii) Technology; iii) Patients' and healthcare professionals' needs; iv) Stakeholders' characteristics; and, v) Financial barriers for further developments. Finally, the most relevant ideas were agreed and clustered into the three dimensions of a priority map (**Figure 4S**): i) Users' satisfaction; ii) Technological viability; iii) Economic viability that were identified as key areas of action to foster prehabilitation scalability and adoption. It was agreed that actions should converge toward the service definition depicted in **Table 1** (second row, third column); that is: "to provide an accessible, round-the-clock personalized and modular service that the patients should be able to use autonomously during the prehabilitation period. The service should combine remotely controlled actions and face to face interactions with health professionals". The attendees agreed on the concept that there was a need for development of agile operational processes aiming at service refinement, using Lean philosophy and tools [2,3]. Overall, five areas for action were formulated: i) Personalization of interventions; ii) Stimulation of a pro-active role of patients, aiming at empowerment for self-management and promotion of physical activity; iii)

Enhanced flexibility of interventions through a highly modular service design; iv) Improved accessibility and logistics; and, v) Achievement of financial sustainability of the services to ensure long-term adoption of cost-effective healthy lifestyles interventions.

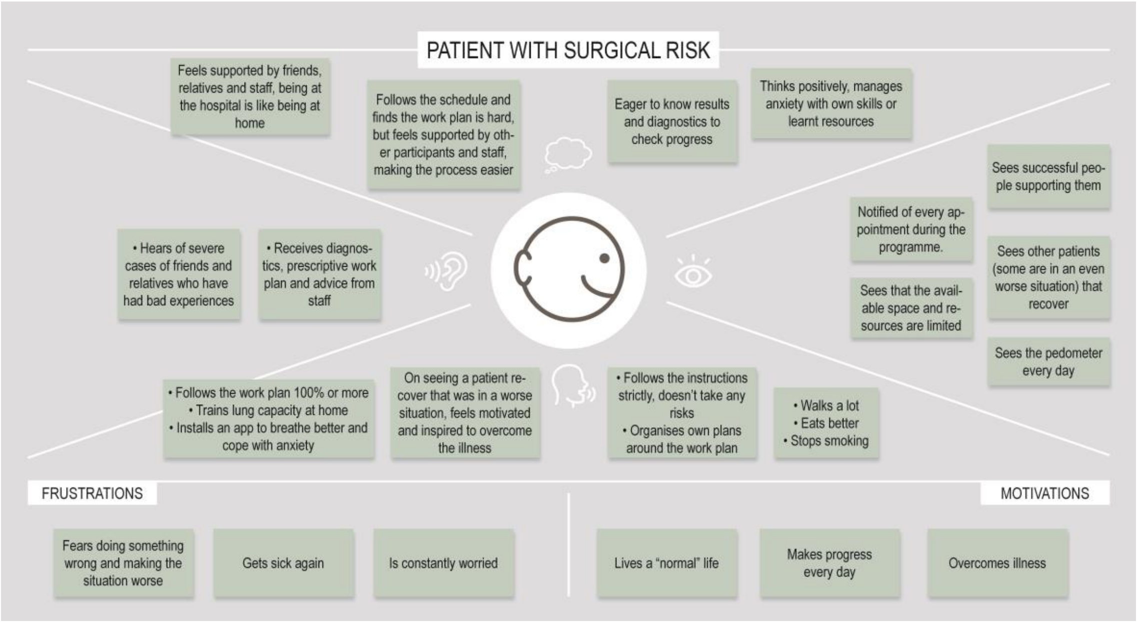

Figure 2S - Empathy map. *The final aim of the empathy map was to have a good understanding about patients enrolled in the prehabilitation service. We empathized with the patients considering the following aspects: i) What they think and feel (upper triangle); ii) What do they see? What is their environment? (right triangle); iii) What do they hear? Who is influencing them? (left triangle); and, iv) What do environment say and how do they act? (down triangle). Moreover we considered their "pains and frustrations" and their "gains and motivations".*

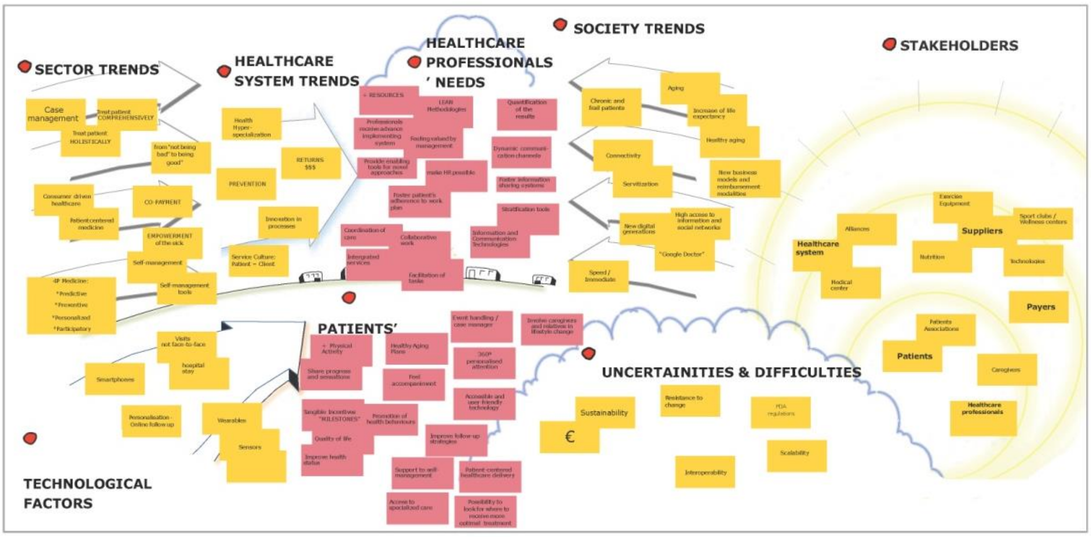

Figure 3S. Context map - *We organized the brainstormed concepts within eight different domains framing the general situation, namely: i) Trends of the healthcare sector; ii) Policy-related trends of the healthcare; iii) General society trends; iv) Technological factors influencing the situation; v) Healthcare professionals needs; vi) Patients' needs; vii) Potential uncertainties and difficulties which can raise during the implementation of the service; and, viii) Different stakeholders of the service. The final aim of the context map was developing a shared big-picture view of the environment of the prehabilitation service to establish a common backdrop for a strategic vision of a complex situation. The general concepts and ideas raised from its debate were finally pooled into a Priority map (**Figure 4S**).*

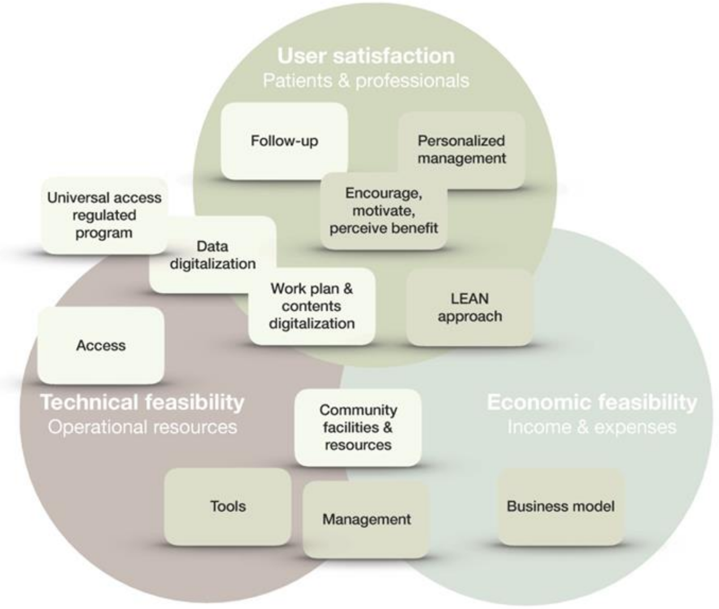

Figure 4S. Priority map. *The figure organizes the relevant factors for a successful scalability of the prehabilitation program in three main domains, namely: i) User satisfaction; ii) Technical feasibility, and, iii) Economic feasibility. Light beige squares contain concepts emerged from the*

*Patient experience map (Fig. 1). Dark beige squares contain concepts emerged from the Context map (**Figure 2S**).*

Session II: Ideation - A total of 44 professionals covering the different profiles attended the second session (**Table 1S**). A first inspirational presentation, of approximate 10 min duration (**Table 1**), updated the audience on the status of the prehabilitation service, its architecture, wireframes, and roadmap for 2017. The second talk, 15 min, was geared towards exploring previous experiences in other fields that have solved the similar challenges. It was followed by ten simultaneous small group creative sessions, 4-5 persons each, that approached the main previously identified challenges under the following success criteria: i) Allow scalability while preserving the quality of the service; ii) Allow reproducibility of the service outcomes in different sites, that is, service transferability; iii) Enhance the adherence of patients to the work plan; iv) Provide key performance indicators to track service effectiveness; v) Foster accessibility to the program; vi) Ensure economic viability for sustainability; and, vii) Conceive the service within a LEAN approach to allow agile implementation and management using minimal resources.

The ideas resulting from the creative sessions were debated by the whole group and then prioritized and pooled into a positioning map (**Figure 5S**). Finally, the ideas incorporated in the positioning map were used to generate a general overview for the refined prehabilitation service workflow to be assessed during the third session.

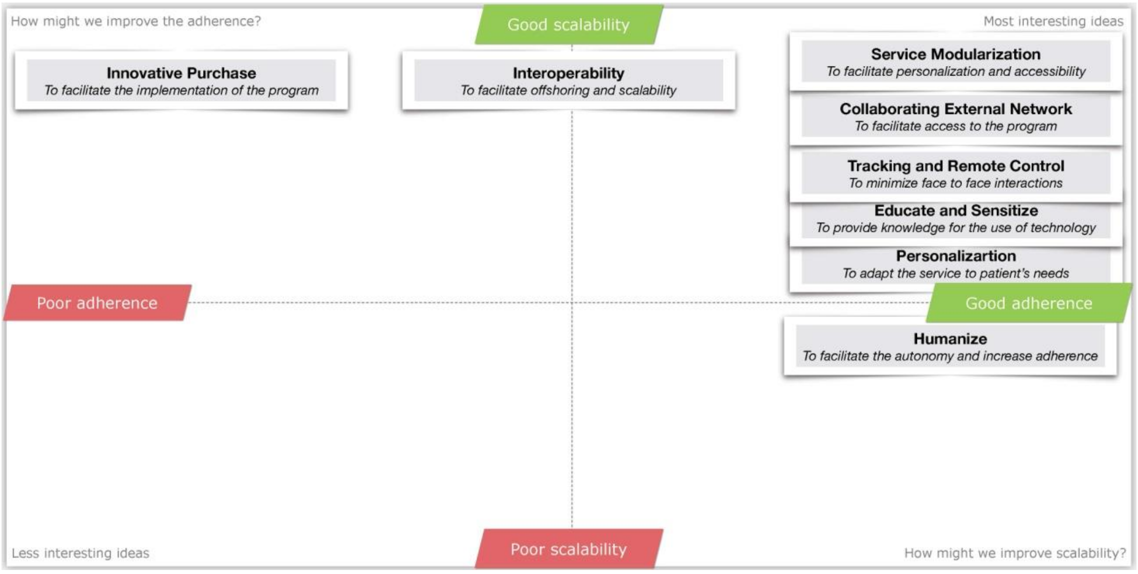

Figure 5S - Positioning map *- This figure shows the best ideas and concepts that emerged from the small creative groups. The ideas were selected in terms of their potential to enhance the patient's adherence to the work plan and their degree of scalability, namely: i) Facilitate innovative purchase to foster the implementation of the service; ii) Promote interoperability among different information systems to promote off-shoring and scalability of the service; iii) Monitor patients by means of wearables to track physical activity and physiological signs in order to enhance patients' adherence to the work plan and foster accessibility and scalability of the service; iv) Provide educational material using information and communication technologies in order to support self-management while fostering the accessibility to the service; v) Generate a capillary network of collaborative wellness and sport centers to enhance accessibility to the service; vi) Personalize the program taking into account patients' characteristics; vii) Promote the humanization of the service by means of a call center to handle events and enhance patients' adherence to the work plan (**Figure 6S**).*

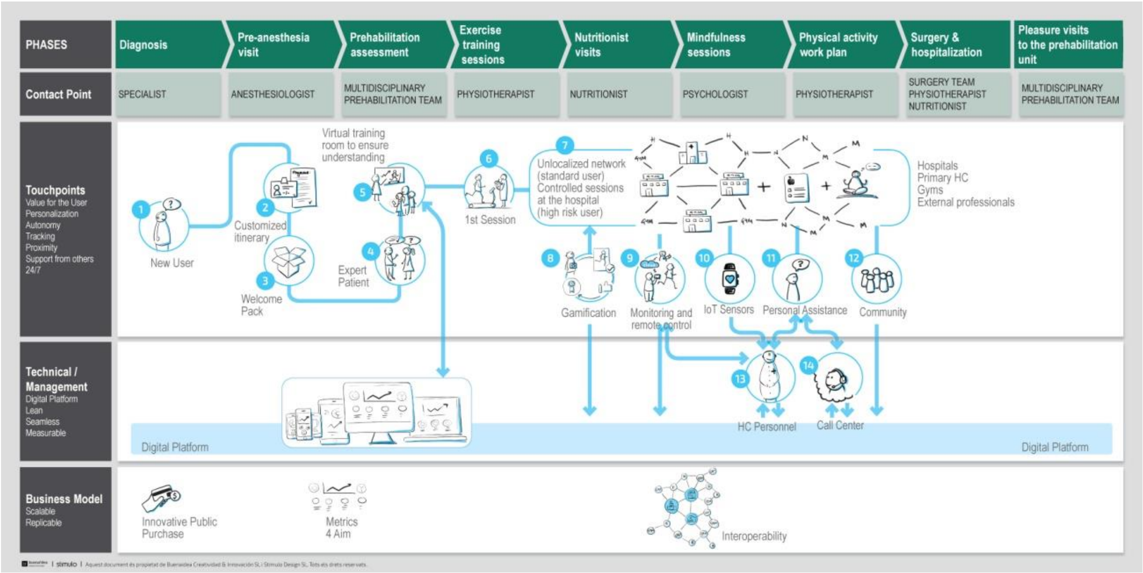

Figure 6S. Workflow of the prehabilitation service.

Session III: Validation - The third session was attended by a total of 43 professionals (**Table 1S**). The categories displayed in the priority map **Figure 4S** were further debated and elaborated in three subgroups of attendees to achieve a well-defined action plan for scalability of the service, as summarized in **Table 1** (fourth row, third column). Briefly, selected actions agreed by the whole group are described below.

Group A – End user touch points. One of the main expectations for the end users was the personalization of the program to be achieved through its modularity. It should consider both biological factors and determinants of adherence to the intervention. Therefore, the subject-specific tailoring of the intervention should envisage the following aspects: i) Aerobic capacity; ii) Nutritional status; iii) Iron reserve profiling at program inclusion; iv) Self-efficacy; v) Facilitators and barriers to physical activity; vi) Psychological & behavioural aspects; and, vii) Logistic factors. Moreover, a key aspect identified by the clinicians was the need for creating a capillary network of community-based health & wellness centres (i.e., primary care, sport centres and gyms) to enhance accessibility. Also, the use of a mobile app, interoperable with the prehabilitation service, allowing tailored remote promotion and monitoring of physical activity (i.e. steps per day and physical activity intensity), patient-reported outcomes (i.e. motivational factors for physical activity, Borg scale and quality of life) and, eventually, other physiological data depending upon patients' characteristics, was considered a highly valuable supporting tool to effectively manage the service in the community setting.

Group B – Technology. To facilitate proper interactions between specialized and community-based actions of the prehabilitation service, as well as to support collaborative work among actors (patients, caregivers, professionals across healthcare tiers), there is a clear need for interoperability of ICT-supporting tools and current hospital information systems. The provision of access to online patient-tailored educational material and to remote support to enable patient empowerment for self-management was highly valued by clinicians.

To fulfil requirements of standardization, flexibility and modularity of the prehabilitation service, an adaptive case management (ACM) system was proposed to support process workflow specification, case management and decision automation [4,5]. The ACM system would provide the required process engine functionality to current hospital information systems. Finally, it was decided that the best solution to enhance end-user adoption was to eliminate complex frontends and drive patient interaction and data collection through an Artificial Intelligence assisted chat (i.e. Chatbot), which would result in a flexible interface closer to the human language [6].

Group C – Business. Preliminary studies on efficacy and costs of the current prehabilitation service at Hospital Clinic de Barcelona [7–9], as well as different randomized controlled trials [10,11], indicate high potential for health value generation both at provider and at health system levels. This suggests that the operational costs of prehabilitation, from the perspective of the hospital, can be fully covered by savings generated through the adoption of the service. Moreover, such savings can be eventually increased by adopting the service refinements elaborated in the DT sessions (i.e., participation of external sport centres, physiotherapy services

companies and use of ICT as enabling tools) (**Table 1**). The group acknowledged that both investments and reimbursement incentives required for service launching may need to be covered by innovative public-private procurement modalities to accelerate the process of scalability of the service. A summary of the DT sessions can be found in (https://stimulo.com/portfolio/prehab/).

Section 1B - 2021 DT sessions

Enclosed find links to the recordings of the two 2021 sessions, 22th June and 13th December: Design thinking session JUNE21 and Design thinking session Dec21, as well as to the final summary report (13th December): final session summary report

Section 2 - Consolidated Framework for Implementation Research (CFIR)

Table 2aS. Reporting of the intervention characteristics within the Consolidated Framework for Implementation Research (CFIR).

<table>
<tr><th>CONSTRUCT</th><th>Implementation at HCB (2016-2019)</th><th>Key recommendations</th></tr>
<tr><td>INTERVENTION CHARACTERISTICS</td><td></td><td></td></tr>
<tr><td>Intervention Source</td><td>• Internally developed.</td><td rowspan="2">• Perioperative care program as an integrated care service</td></tr>
<tr><td>Evidence Strength & Quality</td><td>• Results from internal research (5).</td></tr>
</table>

<table>
<tr><td>Relative Advantage</td><td>• Patients: healthcare outcomes (postoperative complications reduction, aerobic capacity and physical activity enhancement).
• Hospital-based professionals: satisfaction, organizational & clinical results.
• Managers: Cost containment (reduction in intensive care unit stay and 30-day hospital readmissions).</td><td rowspan="6">• Core components:
1) High-intensity exercise training
2) Behavioural cognitive therapy
3) Nutritional support
4) Service workflow defined
5) Define target patients' profiles
6) Structure to support the workflow

• Adaptability of non-core components is required.

• Continuous quantitative & qualitative build-in evaluation is needed.</td></tr>
<tr><td>Adaptability</td><td>• Core components: i) High-intensity exercise training; ii) Behavioural cognitive therapy; iii) Nutritional support; iii) Workflow defined & patient profiles; iv) Structure to support the workflow.
• Adaptable components: i) Hospital or community-based exercise training; ii) Anxiety/depression management; iii) Haemoglobin optimization; iv) Smoking cessation program.</td></tr>
<tr><td>Trialability</td><td>• Results from internal research (5, 7).
• A building-blocks strategy with stepwise progression of deployment with continuous evaluation (8).</td></tr>
<tr><td>Complexity</td><td>• High complexity process requiring coordination of: i) Clinical protocols; ii) Redefine tasks & roles; iii) Community-based logistics; iv) Digital support; v) Professionals' training; vi) Information for patients; vi) Coordination among community and hospital settings; vii) Reimbursement modalities.</td></tr>
<tr><td>Design Quality & Packaging</td><td>• Key elements: i) Early detection of candidates in order to maximize the prehabilitation period; ii) Comprehensive explanation of the program to patients and supporting healthcare professionals; and, iii) Coordination of the interventions within the program to optimize the number of patients' displacements to the hospital.</td></tr>
<tr><td>Cost</td><td>• Implementation costs were covered by efficiencies generated without allocation of a specific budget for this purpose.
• Costs from the hospital perspective were calculated showing health-value generation for major digestive surgery (7)</td></tr>
<tr><td>Patients' needs & resources</td><td>• Alignment with PRISM (Patient-centred care): i) Patients' preferences, facilitators & barriers taken into account (7); ii) Lean strategy minimizing costs; and, iii) Patients' satisfaction & opinions considered and taken as inputs to improve.</td><td rowspan="2">• Patient-centred orientation should be a core trait.

• Networking across experiences enriches the programs.

• Site customization is required to minimize potential negative impacts of external factors.</td></tr>
<tr><td>Cosmopolitanism</td><td>• Build-up as a functional integration of healthcare providers at district level (AISBE) (9)
• Progress through continuous interactions among regional providers (10)
• Leading role at EU level (four stars reference site EIP-AHA) (11).</td></tr>
</table>

**Pier pressure**	• Support from the single-public payer and internal managers.<br>• Moderate transient resistances from professionals from primary care and other internal clinical units.<br>• Expansion beyond HCB limited by reimbursement modalities to other health-district provider organizations.	
**External Policy & Incentives**	• Implementation was an internal decision with some external support from the industry.<br>• Program should be considered as a learning experience.<br>• EU funding provided additional financial support (12).	
**Readiness for implementation**	• Readiness for implementation at regional level and at HCB level (13).<br>• Leaders' engagement varies among hospital institutes. Champion-driven initiative.<br>• Creation of the prehabilitation unit at HCB.<br>• Lack of technological platform and administrative support for the program.	

Table 2bS. Reporting of the outer setting characteristics within the Consolidated Framework for Implementation Research (CFIR).

<table>
<tr><th>CONSTRUCT</th><th>Implementation at HCB (2016-2019)</th><th>Key recommendations</th></tr>
<tr><td>OUTTER SETTING</td><td></td><td></td></tr>
<tr><td>Patients' needs & resources</td><td>• Alignment with PRISM (Patient-centred care): i) Patients' preferences, facilitators & barriers taken into account; ii) Lean strategy minimizing costs; and, iii) Patients' satisfaction & opinions considered and taken as inputs to improve.</td><td rowspan="5">• Patient-centred orientation should be a core trait.
• Networking across experiences enriches the programs.
• Site customization is required to minimize potential negative impacts of external factors.</td></tr>
<tr><td>Cosmopolitanism</td><td>• Progress through continuous interactions with other international teams promoting prehabilitation as mainstream service within the frame of the International Prehabilitation Society (https://prehabsociety.com/). Chair of the International Congress on 28/29 May 2020 in Barcelona.
• Regular interactions with international teams within the frame of EU projects: EIP-AHA, EIT-Health.</td></tr>
<tr><td>Pier pressure</td><td>• No external competitive pressure from other organizations.
• Moderate professional resistances within the organization.</td></tr>
<tr><td>External Policy & incentives</td><td>• Implementation was an internal decision with some external support from the industry.
• Program should be considered as a learning experience.
• EU funding provided additional financial support (H2020; EIT Health).
• Expansion limited due to lack of specific reimbursement incentives</td></tr>
<tr><td>Readiness for implementation</td><td>• Readiness for implementation at regional level (13) and at HCB level.
• Leaders' engagement varies among hospital institutes. Champion-driven initiative.
• Creation of the prehabilitation unit at HCB.
• Lack of technological platform and administrative support for the program.</td></tr>
</table>

Table 2cS. Reporting of the inner setting characteristics within the Consolidated Framework for Implementation Research (CFIR).

<table>
<tr><th>CONSTRUCT</th><th>Implementation at HCB (2016-2019)</th><th>Key recommendations</th></tr>
<tr><td>INNER SETTING</td><td></td><td></td></tr>
<tr><td>Structure Characteristics</td><td>• Institutional traits favouring the driving role: i) Needs for reduction in hospital length of stay and postoperative complications; ii) Leading role of professionals in the management; iii) Institutional choice towards continuum of care (14); and, iv) Digital transformation in place.
• The vertical organization of HCB in clinical institutes was a relative barrier.</td><td rowspan="5">• Bottom-up & top-down interactions are needed for success.

• Key resources to generate and reinforce a positive climate change are needed.</td></tr>
<tr><td>Networks & Communications</td><td>• High prehabilitation professional’s engagement & great acceptation of the program from healthcare professionals outside the prehabilitation unit.
• The immaturity of the technology supporting program management was an important barrier.</td></tr>
<tr><td>Culture</td><td>• Mix of type 1 (team culture) and type 3 (entrepreneurial culture) with high engagement of professionals.</td></tr>
<tr><td>Implementation Climate</td><td>• Positive climate for entrepreneurship among stakeholders despite acknowledgment of some internal peer resistances.
• Tension for change was limited to implementation leaders (champions) supported by management.
• Program acknowledged as fully aligned with the institutional healthcare delivery values and policies.
• Implementation priority varies among hospital institutes.
• No incentives & rewards were planned.
• Goals were clearly defined and assessed by PDSA cycles. Feedback provided was well accepted.
• Positive acceptance from professionals in the development of new interventions to improve patient-centred care.</td></tr>
<tr><td>Readiness for implementation</td><td>• Readiness for implementation at regional level (13) and at HCB level.
• Leaders’ engagement varies among hospital institutes. Champion-driven initiative.
• Creation of the prehabilitation unit at HCB.
• Lack of technological platform and administrative support for the program.</td></tr>
</table>

Table 2dS. Reporting of the characteristics of individuals within the Consolidated Framework for Implementation Research (CFIR).

<table>
<tr><th>CONSTRUCT</th><th>Implementation at HCB (2016-2019)</th><th>Key recommendations</th></tr>
<tr><td>CHARACTERISTICS OF INDIVIDUALS</td><td></td><td></td></tr>
<tr><td>Knowledge & Believes about the intervention</td><td>• Sustained positive perception of the intervention by both patients and healthcare professionals.
• Acknowledgement of enhanced healthcare outcomes, reduction of healthcare resources’ use and positive impact on costs.
• Passive resistance of small sectors of professionals.</td><td rowspan="5">• Continuous monitoring of satisfaction levels and consideration of feedback from patients and professionals is highly recommended (15).</td></tr>
<tr><td>Self-efficacy</td><td>• One of the main components of the program is a motivational intervention fostering self-efficacy for long-term healthy lifestyles (i.e., physical activity, nutrition and toxic habits).</td></tr>
<tr><td>Individual stage of change</td><td>• Slow progressive achievement of active engagement of stakeholders throughout the unit consolidation process at HCB.</td></tr>
<tr><td>Individual identification with organization</td><td>• A culture of individual identification with unique traits of the HCB’s organization (health professionals’ involvement in management) facilitated implementation.</td></tr>
<tr><td>Other personal attributes</td><td>• Tolerance, motivation, innovativeness, and learning style have been reinforced during the implementation process.</td></tr>
</table>

Table 2eS. Reporting of the process characteristics within the Consolidated Framework for Implementation Research (CFIR).

<table>
<tr><th>CONSTRUCT</th><th>Implementation at HCB (2016-2019)</th><th>Key recommendations</th></tr>
<tr><td>PROCESS</td><td></td><td></td></tr>
<tr><td>Planning</td><td>• The deployment plan organized by blocks at two different levels: i) Specific patients’ profiles of the clinical institutes at HCB; and, ii) Implementation of new tailored interventions within the prehabilitation program. The plan was progressively implemented with adaptations considering both available resources and feedbacks received from professionals and patients.</td><td rowspan="4">• A building-blocks implementation strategy, with appropriate site customization prioritizing engagement, is required.
• Continuous evaluation of results.</td></tr>
<tr><td>Engaging</td><td>• Champions triggered and conducted deployment with support, and direct interactions, with HCB’s management.
• A specific group of healthcare professionals with high degree of commitment contributed to consolidation.</td></tr>
<tr><td>Executing</td><td>• Previous experience with a RCT (5, 7) helped to set the basis of the deployment plan which was executed and enriched during consolidation process.
• Continuous monitoring and adaptability of the implementation process were key elements for successful adoption (5, 7, 15).</td></tr>
<tr><td>Reflecting & Evaluating</td><td>• Continuous quantitative & qualitative assessments were done and reported (15).
• Internal monthly meetings and periodic reporting to HCB’s executive committee were scheduled and done.</td></tr>
</table>

Section 3 - Key Performance Indicators (KPI)

Table 3S. Key performance indicators for long-term monitoring of the prehabilitation service.

DOMAIN	INDICATORS	MEASUREMENT
**Structure**	**Coverage**	• **Numerator:** Total of patients included in prehabilitation.<br>• **Denominator:** Total of target patients.
**Process**	**Rate of dropouts**	• **Numerator:** Total of patients discharged from prehabilitation.<br>• **Denominator:** Total of patients included in prehabilitation.
	**Rate of adherence**	• **Numerator:** Total cumulative number of sessions of prehabilitation attended.<br>• **Denominator:** Total cumulative number of sessions of prehabilitation offered.
	**Quality assurance program scoring**	• **Numerator:** Quality assurance program scoring *100.<br>• **Denominator:** 10.
**Outcome**	**Comprehensive complication index** (CCI) **at 30 days**	• **Numerator**: Total cumulative postoperative CCI at 30 days of the patients discharged from prehabilitation.<br>• **Denominator**: Total number of patients discharged from prehabilitation and underwent surgery.
	**Hospital length of stay** (HLoS)	• **Numerator:** Total cumulative postoperative HLoS of the patients discharged from prehabilitation.<br>• **Denominator:** Total number of patients discharged from prehabilitation and underwent surgery.
	**Use of healthcare resources at 30 days** (HCR30d)	• **Numerator:** Use of HCR30d of patients discharged from prehabilitation.<br>• **Denominator:** Total number of patients discharged from prehabilitation and underwent surgery.

CCI: comprehensive complications index; SPC: standard preoperative care; HLoS: hospital length of stay; HCR30d: Healthcare resources at 30 days.

References

1. Schonenberg H, Nielsen E, Syse T, Bescos C. Experiences on scaling Care Coordination and Telehealth Best Pratices Handbook. 2019;1–59. Available from: https://www.philips.com/c-dam/corporate/newscenter/global/standard/resources/healthcare/2019/act-at-scale/ACT-at-Scale_Handbook.download.pdf)
2. Coletta A. The Lean 3P advantage. A practitioner's guide to the production preparation process. Productivity Press; 2012.
3. Hicks C, McGovern T, Prior G, Smith I. Applying lean principles to the design of healthcare facilities. Int J Prod Econ [Internet] 2015;170:677–686. [doi: https://doi.org/10.1016/j.ijpe.2015.05.029]
4. Swenson K. Mastering the Unpredictable [Internet]. Uma ética para quantos? 2012. p. 81–87. PMID:15003161
5. Herrmann C, Kurz M. Adaptive Case Management: Supporting Knowledge Intensive Processes with IT Systems BT - S-BPM ONE - Learning by Doing - Doing by Learning. In: Schmidt W, editor. Berlin, Heidelberg: Springer Berlin Heidelberg; 2011. p. 80–97.
6. Dahiya M. A Tool of Conversation: Chatbot. Int J Comput Sci Engenieering [Internet] 2017;5(5):158–161. Available from: www.ijcseonline.org
7. Barberan-Garcia A, Ubré M, Roca J, Lacy AM, Burgos F, Risco R, Momblán D, Balust J, Blanco I, Martínez-Pallí G. Personalised Prehabilitation in High-risk Patients Undergoing Elective Major Abdominal Surgery : A Randomized Blinded Controlled Trial. Ann Surg 2018;267(1):50–56. PMID:28489682
8. Barberan-Garcia A, Gimeno-Santos E, Blanco I, Cano I, Martínez-Pallí G, Burgos F, Miralles F, Coca M, Murillo S, Sanz M, Steblin A, Ubré M, Benavent J, Vidal J, Sitges M, Roca J. Protocol for regional implementation of collaborative self-management services to promote physical activity. BMC Health Serv Res [Internet] 2018;18(1):560. PMID:30016944
9. Barberan-Garcia A, Ubre M, Pascual-Argente N, Risco R, Faner J, Balust J, Lacy AM, Puig-Junoy J, Roca J, Martinez-Palli G. Post-discharge impact and cost-consequence analysis of prehabilitation in high-risk patients undergoing major abdominal surgery: secondary results from a randomised controlled trial. Br J Anaesth 2019 Oct;123(4):450–456. [doi: 10.1016/j.bja.2019.05.032]
10. Arthur HM, Daniels C, McKelvie R, Hirsh J, Rush B. Effect of a preoperative intervention on preoperative and postoperative outcomes in low-risk patients awaiting elective coronary artery bypass graft surgery. A randomized, controlled trial. Ann Intern Med 2000 Aug;133(4):253–62. PMID:10929164
11. Barakat HM, Shahin Y, Khan JA, McCollum PT, Chetter IC. Preoperative Supervised Exercise Improves Outcomes After Elective Abdominal Aortic Aneurysm Repair: A Randomized Controlled Trial. Ann Surg 2016;264(1):47–53. PMID:26756766
